# Supplementary material for: Ruminal fermentation, microbial population and lipid metabolism in gastrointestinal nematode-infected lambs fed a diet supplemented with herbal mixtures
Source: PLoS One. 2020 Apr 16;15(4):e0231516. doi: 10.1371/journal.pone.0231516 (PMC7161954; doi:10.1371/journal.pone.0231516)
Supplement: Attachment — Submitted filename: Answer to Editor and Reviewers- Herbmix-LS 1.2.20 v2.doc [file pone.0231516.s003.doc]

Poznan, 11.03.2020

Simon Russell Clegg, PhD
Academic Editor
PLOS ONE

Manuscript ID: PONE-D-19-31838 Ruminal fermentation, microbial population and lipid metabolism in gastrointestinal nematode-infected lambs fed a diet supplemented with herbal mixtures
PLOS ONE

Dear Editor

Dear Reviewers

Thank you very much for evaluating again our manuscript and minor suggestions. We follow your instruction and we marked our changes as follows:

- yellow marks words that have been changed, added, or moved to a better location

- the **bold text** was used for the comments of Referees

-AU: stands for our replies and comments

**We would like to thank the Referees for all the valuable comments and suggestions that helped us to improve our manuscript.**

**We have introduced all changes indicated in the Editor and Reviewers’ comments and concerns.**

Please use the space provided to explain your answers to the questions above. You may also include additional comments for the author, including concerns about dual publication, research ethics, or publication ethics. (Please upload your review as an attachment if it exceeds 20,000 characters)

**Reviewer #2: Even though, authors have been revised accordingly to my comments, there are minor point need more address.**
**L82-83: provide detail mechanism how polyphenols inhibit the populations of microbes.**

AU: Thank you for your suggestion. We added the following information L82-85:

*Polyphenols inhibit the populations and/or activity of microbes responsible for methanogenesis and biohydrogenation by among others changing the rumen environment (pH value) and through the toxic effect on methanogens, consequently lowering methane emission and biohydrogenation rate of UFA in the rumen.*

**L143-144: what kind of feed was fed to lamb? Detail.**

AU: In our opinion all detailed information regarding lambs feeding are located in L 118-121

*Animals were fed a concentrate mixture (500 g dry matter (DM)/d), herbal mixtures (non-commercial mixtures - Mix1 and Mix2; 100 g DM/d) and meadow hay (ad libitum). The concentrate mixture was composed of 700 g/kg of barley, 220 g/kg of soybean meal, 48 g/kg of wheat bran, 5 g/kg of bicarbonate and 27 g/kg of mineral-vitamin premix.*

Because of it we would like kindly to ask the Reviewer to keep as it is.

**L671: Conclusion is too hard understand, please revise and make to related to hypothesis and objective study.**

AU: Thank you for your suggestion. We improved our conclusion, and we hope you will accept it.

Now the conclusion is as follow:

L 674-683

*Infection did not elicit major impacts on the ruminal fermentation characteristics and FA profiles in tissues, but it increased TBARS in serum and meat after storage. Herbal mixtures supplementation had no effect on the ruminal fermentation characteristics including the ruminal methane production, but increased total VFA concentrations and DM digestibility in vitro. Supplementation of herbal mixtures to the diets of GIN parasite infected-lambs decreased MCFA and increased LCFA in liver and meat, and decreased lipid oxidation in meat due to their inhibitory effects on the ruminal biohydrogenation. From this result and previous results [17], it can be concluded that Mix1 may reduce parasitic burdens as well as improve LCFA proportion and oxidative stability in meat, which may prove win-win situations in ruminant production.*

Reviewer #3: I wish to thank the authors for their detailed review response to the comments which I made on the last manuscript. I know many were very minor, and thank you for addressing them, and the comments to the others which are all acceptable to me (most were asked out of curiosity).
I have made a few very minor comments, which if addressed, I would not expect to see a revised version prior to publication.
Thanks once again, and best wishes

**Line 107- remove were obtained from the same farm- as you say that twice**

AU: Thank you for your correction. It has been done.

**Line 173- after the adaptive period**

AU: It was corrected. Thank you.

**Line 303- you may wish to say if any of these genes were sequenced as part of the PCR development**

AU: We are sorry for misunderstanding. Transcript expression of the 5 genes investigated in this study was carried out with the RT-qPCR method. Any of the sequencing procedure has not been applied.

**Line 571- not found differences doesn’t make sense- maybe no differences were found?**

AU: It was improved. Thank you. **Line 575- comma after study may make this sentence a bit easier to read**

AU: Thank you – you are right. We introduced necessary changes into the following sentence:

*The methane production which showed no differences both in in vitro and in vivo by Mix1 or Mix2 confirmed the results of the previous study, which presented the interaction of S. officinalis basic components and phytochemical compounds causing the reduced antimethanogenic activity due to lower availability of substances for microorganisms [46].*

**Disucssion- I believe that Archaea needs capitalising throughout**

AU: It was corrected. Thank you.

**We would like once more to thank very much the Editor and Reviewers for all the valuable comments and suggestions that helped us to improve our evaluated manuscript.**
